# Affordable Portable Platform for Classic Photometry and Low-Cost Determination of Cholinesterase Activity

**DOI:** 10.3390/bios13060599

**Published:** 2023-05-31

**Authors:** Ondřej Keresteš, Miroslav Pohanka

**Affiliations:** Faculty of Military Health Sciences, University of Defence, CZ-50001 Hradec Kralove, Czech Republic; miroslav.pohanka@unob.cz

**Keywords:** field-analysis, open-source, photometry, point-of-care test, affordable, cholinesterase, pesticide

## Abstract

Excessive use of pesticides could potentially harm the environment for a long time. The reason for this is that the banned pesticide is still likely to be used incorrectly. Carbofuran and other banned pesticides that remain in the environment may also have a negative effect on human beings. In order to provide a better chance for effective environmental screening, this thesis describes a prototype of a photometer tested with cholinesterase to potentially detect pesticides in the environment. The open-source portable photodetection platform uses a color-programmable red, green and blue light-emitting diode (RGB LED) as a light source and a TSL230R light frequency sensor. Acetylcholinesterase from *Electrophorus electricus* (AChE) with high similarity to human AChE was used for biorecognition. The Ellman method was selected as a standard method. Two analytical approaches were applied: (1) subtraction of the output values after a certain period of time and (2) comparison of the slope values of the linear trend. The optimal preincubation time for carbofuran with AChE was 7 min. The limits of detection for carbofuran were 6.3 nmol/L for the kinetic assay and 13.5 nmol/L for the endpoint assay. The paper demonstrates that the open alternative for commercial photometry is equivalent. The concept based on the OS3P/OS3P could be used as a large-scale screening system.

## 1. Introduction

As agriculture expands and demands for higher yields without sacrificing quality increase, plants must be treated before signs of insect, fungal or other diseases emerge [1]. Although organophosphate and carbamate pesticides are widely used pesticides, both groups of these substances act as cholinesterase inhibitors, making them harmful. This is why these substances have been regulated [2].

Acetylcholinesterase (AChE) has been selected as a commonly used biorecognition element for the construction of an advanced biosensor system. Functional AChE is expressed in the central and peripheral nervous systems and provides smooth neurotransmission by hydrolyzing acetylcholine, making it an essential enzyme for memory and learning. AChE inactivity can cause organ failure or even death [3,4]. The AChE of the electric eel was chosen because of its high degree of similarity to the human AChE [5]. Using acetylcholinesterase as a biorecognition element is a common approach to the selective determination of pesticides.

Although the occurrence of a possible change in AChE activity after inhibition by various compounds is important for safety reasons [6], there are other important cases of the use of cholinesterase inhibitors: pesticide detection [7,8] and pharmaceutical screening [3,9,10].

Carbofuran, a representative of the carbamate inhibitors, is a regulated pesticide in the European Union. However, it can still be found in nature despite the regulation [11,12]. 

The introduction of new detection methods is the primary goal for controlling environmental pollution, which is increasing due to the massive use of chemicals in nature and additional non-target effects [13,14].

Point-of-care testing (POCT) is advantageous in areas where analyte or matrix degradation may occur and prolonged transport may compromise the efficiency of the laboratory method. This is the reason for monitoring the analytes close to the sampling site. Thus, pesticides could be detected efficiently in the field.

In the literature, there are stand-alone photometers suitable for field measurement, which are mainly 3D-printed [15,16,17,18] or made from wooden plates cut by a laser beam [19]. There are also smartphone adapters that use the light sensor on the front of. The cell phone [20]. Adapters for luminescence and fluorimetry have also been described [18,21,22]. Although efficient dual-reading fluorometers are described in the recent literature [15,23], an instrument tailored for specific requirements, such as the one proposed in this paper, is not yet available. This novel platform could rapidly reduce the budget for relevant studies with enzyme-based biosensors in liquid samples, e.g., for water analysis. After miniaturization, it could also be used as a detector for low-cost flow-through systems.

There are several publications on cholinesterase-based field sensors. However, electrochemistry is the commonly used principle [24,25,26,27,28,29,30], and there are a few works proposing optical biosensors for cholinesterase activity [31], even with low-cost electronics [32]. Concerns about the validation of optical biosensors for field analysis provide a reason to transfer standard photometry to the field instead of validating novel experimental methods [33].

The application of the Ellman method is the determination of cholinesterase inhibitors [34,35,36,37]. It has been applied to many different platforms, paper-based colorimetry [38,39,40,41], or microplate reading [42].

Although this is a standard method for the testing of cholinesterase, it is very difficult to find open-source systems tested for using this assay. Not many systems have been tested for kinetic measurement. Apart from the analysis of nitrate in water with the system using a similar sensor [18], it is not possible to say how the response of the named sensors to changing environments has been investigated in real time.

In this work, the Open-source portable photodetection platform (OS3P) is proposed as a response to the gap. It is a system based on open-source electronics. The main components are an Arduino UNO Rev 3 microcontroller board, a sensitive light frequency sensor TSL230R, and an red, green and blue light-emitting diode (RGB LED) module with a common anode. The total cost of this analyzer is about USD 100. The program that controls the system can change the color based on the desired wavelength of the absorption maxima of the target analyte. Color changes can be made in the Data Streamer interface while the analysis is running. OS3P has been tested by running kinetic and end-point regimes of the Ellman assay.

## 2. Materials and Methods

### 2.1. Preparation of the Open-Source Portable Photodetection Platform (OS3P)

#### 2.1.1. 3D Printing

The 3D design of OS3P was made in Fusion 360 v.2.0.16009 (Autodesk, San Rafael, CA, USA). The 3D model was cut and processed for printing with PrusaSlicer and 3D-printed with Prusa Mini+ (Prusa Research, Prague, Czech Republic). The OS3P box was printed from white and black ASA polymer (acrylonitrile styrene acrylate, Prusa Research, Prague, Czech Republic). The system was assembled using an Arduino UNO R3 (Arduino, New York City, NY, USA), TSL230R photodiode sensor (TAOS, Texas Advanced Optoelectronic Solutions, TX, USA), and red, green, blue light-emitting diode (RGB LED module KS0032 (Keyestudio, Shenzhen, China).

#### 2.1.2. Design of the Sensor System

The electronics for the OS3P were assembled from a 3D-printed model made by the authors and a 3D-printed analog of the breadboard holder [43] and Arduino UNO and the breadboard obtained from Arduino and supplied with the Arduino Starter Kit. The light-frequency sensor TSL230R [44] was mounted on the breadboard and solderless connected to the Arduino UNO R3 board using jumper wires. The RGB LED module KS0032 [45] was connected to the Arduino board using Dupont wires, which are commonly used for solderless connections in prototyping.

**Figure 1:**
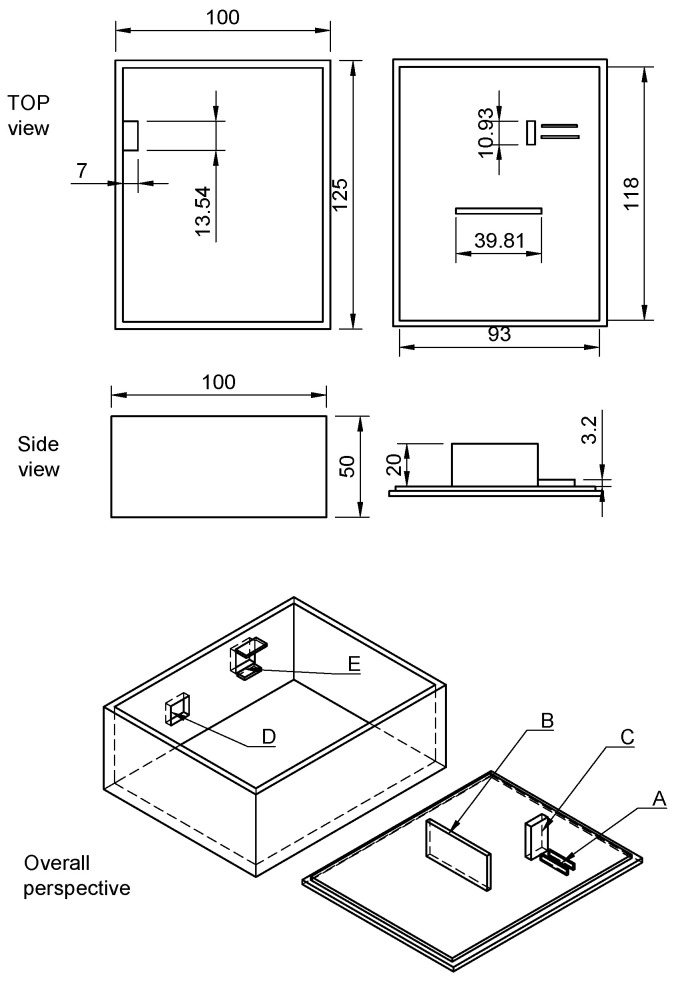
Schematic of OS3P box with inner parts placement—(“A”) LED module holder. (“B”) Cover of parasitic light from the LED on the Arduino UNO R3 board. (“C”) Cuvette stabilizer. It is used to stabilize the standard position of the cuvette and prevent the cuvette from failing. (“D”) Hole for USB-B connector. (“E”) Cuvette hole with reinforced wall for better cuvette insertion.

**Figure 2:**
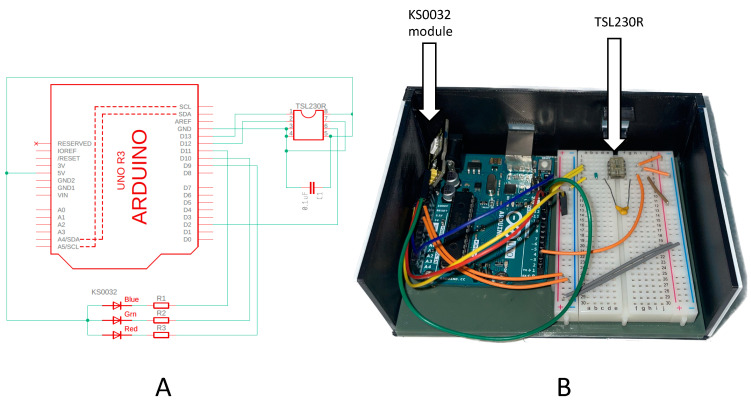
(**A**) Schematic of an Open-source portable photodetection platform (OS3P). (**B**) Overview of the connected hardware.

The dimensions of the 3-D printed box are 125 mm (L) × 100 mm (W) × 50 mm (H), see Figure 1. The LED module was glued to the lid of the box with ABS-juice in the appropriate opposite direction to the TSL230R mounted on the breadboard (“A”). The wiring of TSL230R was inspired by the Meyer tutorial example [46] and the general wiring scheme is shown in Figure 2. 

Arduino UNO has several LEDs on it that light up during operation; therefore, to prevent light impurities, a wall (“B”) was created to cover the space between the microcontroller and the breadboard when the lid is on.

**Figure 3:**
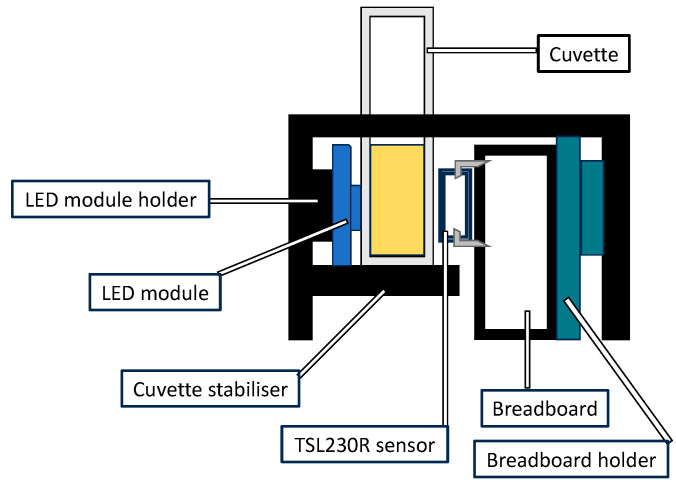
Cutaway view of the OS3P measuring aperture after box assembly.

The LED module was wide, and it was necessary to ensure that the cuvette would not fall through; for that reason, the handle (“C”) was added to the lid to stop the cuvette from sliding in. A diagram of placing the electronic parts is shown in Figure 3. 

### 2.2. Software Controlling the OS3P

The Arduino IDE v2.0.1 software (Arduino, New York City, NY, USA) was first used to thoroughly program and control the OS3P thorough the provided firmware (OS3P.ino [47]).

The Arduino Uno has several digital pins that can be used to connect one or even more LEDs.

The RGB LED requires 3 digital pins with optional pulse-width modulation (PWM) pins. In this work, since the RGB module must be controlled by 3 separate signals of PWM pins with values between 0 and 255, the desired wavelength (400–700 nm) must have been recalculated for each channel to emit an equivalent portion of intensity. A library named <WavelenghtToRGB> was used to calculate this combination to emit the chosen color mix. This library must be included in the firmware. It is important to know that the user will want to use LEDs with a common anode or common cathode. It is necessary to choose the right answer to the bool value “Common anode”.

The TSL230R sensor can be optimized with thorough sensitivity setting by controlling the S0 and S1 pins on the sensor thorough HIGH/LOW values in OS3P_setup function in firmware. The value of “TSL230_samples” can be changed in the first part of the code. The higher the value, the more pulses are integrated and the lower the response rate. All measurements were made in high sensitivity mode. This mode can be changed in the OS3P.ino firmware, also as “TSL230_sample” values.

In operating mode, the OS3P can be controlled by sending single letter commands by serial communication response (+/− for modulation color by the defined value “shift”, E for immediate setting color for Ellman assay).

### 2.3. Light Source Characterization

**Figure 4:**
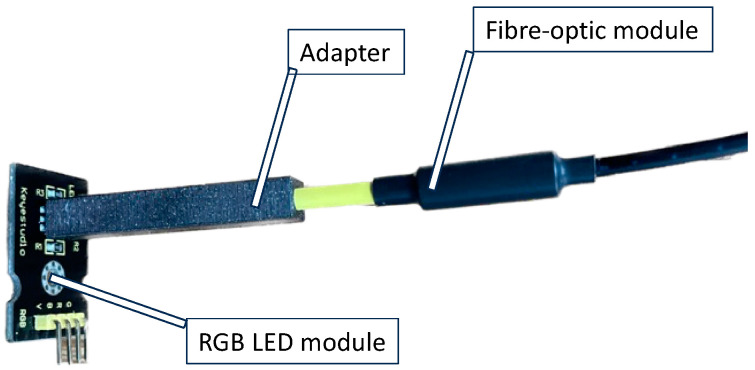
Emission aperture details. An optical fiber connected to Spectrovis Plus was used. The red, green and blue light-emitting diode (RGB LED) module was controlled by Arduino UNO and set up for maximum intensity.

The emission of the RGB LED module was measured with the Spectrovis Plus fiber optic module (Vernier, Beaverton, OR, USA). The light intensity of each color channel was measured in fluorescence mode with Spectrovis Plus in the Logger Pro v3.16.2 software (Vernier, Beaverton, OR, USA). An additional 3D-printed adapter [47] was used to establish the optical path between the RGB LED module and the fiber optic module. It is shown in Figure 4.

### 2.4. Testing of the TSL230R Sensor

To obtain the lowest possible sensor response, a cuvette filled with distilled water was inserted into OS3P and covered with a 3D-printed plastic cap. The system was therefore connected to computer. Ten output frequency values were obtained using the Data Streamer add-in. The mean and standard deviation were calculated for ten replicates. The testing of different light conditions was done by evaluating at least 30 response values for each color.

### 2.5. Measement of Color Solutions

Phenol red (Thermo Scientific, Watham, MA, USA) was measured using two different colors. First, the calibration curve of solutions diluted with phosphate buffer saline (pH = 7.4) was evaluated in two series of 50 mmol/L. Dilution of the solutions was stopped when the color change between the two lowest concentrations was insignificant. The frequency output values were measured in triplicate and as a mean of at least 10 values in the procedure. All experimental data were acquired using, Microsoft Excel v2302 (MS Excel), with a data streamer add-in (Microsoft, Redmond, WA, USA).

### 2.6. Preparation of Enzyme Concentrate

The AChE (EC 3.1.1.7) from *Electrophorus electricus* (Merck, Darmstadt, Germany) was selected as the enzyme for the determination of carbofuran (Merck, Darmstadt, Germany) as a model inhibitor of cholinesterase. The enzyme standard sample was prepared by dissolving the crude enzyme protein in phosphate-buffered saline (pH 7.4, PBS; Merck, Darmstadt, Germany), with the addition of bovine serum albumin (Merck, Darmstadt, Germany) added as a stabilizing agent at a total concentration of 0.5 mg/mL (w/V). The activity of this concentrate was optimized to provide an enzymatic reaction with 0.5–1 OD after 2–5 min of incubation. This mixture was dispensed into microtubes and stored in the freezer (−20 °C).

### 2.7. Enzyme Assay—Optimizing the Effective Substrate Concentration

The Ellman’s assay for cholinesterase activity is based on the reaction of thiocholine (Merck, Darmstadt, Germany) and 5,5′-Dithio-bis-(2-nitrobenzoic Acid) (DTNB; Merck, Darmstadt, Germany), which forms 2-Nitro-5-thiobenzoic acid (TNB) [34]. In a standard microcuvette, 400 μL of DTNB (1 mmol/L) in PBS, 25 μL of AChE concentrate, 25 μL of 2-propanol, and 450 μL of PBS were mixed. The reaction was initiated by the addition of acetylthiocholine chloride (ATChCl, Merck, Darmstadt, Germany). The time difference used for the evaluation was 5 min.

The substrate for the reaction was diluted in a twofold series to a concentration of 0.03125–2 mmol/L. The enzyme activity was determined using two methods. The first was to evaluate the slope of the curve during the ongoing biochemical reaction and the second was to subtract the lower value from the higher value of the frequency measurement.

### 2.8. Enzyme Assay Conducted by OS3P

In a standard microcuvette, 400 μL of DTNB in PBS (1 mmol/L), 25 μL of AChE concentrate, 25 μL of 2-propanol, and 450 μL of PBS were mixed. The reaction was started by adding 100 μL of acetylthiocholine chloride (10 mmol/L, ATChCl, Merck, Darmstadt, Germany). A frequency change was observed for 6 min. The response rate was greater than 2 values/s. The measurement was carried out in triplicate.

### 2.9. AChE Inhibitor Assay

(a) The optimal time for all molecules of the inhibitor to bind to AChE.

The reaction mixture is similar to that used in the substrate optimization study. Pure 2-propanol (Merck, Darmstadt, Germany) was used as a negative control additive and carbofuran solution in 2-propanol was tested. The use of 2-propanol is advantageous due to low decrease in AChE activity using this solvent at 5% (V/V) concentration. The final concentration of carbofuran in the cuvette was 0.5 μmol/L. After obtaining the maximum inhibitory potential of carbofuran, 10–15% residual activity should be observed. The reaction was started by adding the enzyme the last, initially to obtain the preincubation interval of 0 s. The interval was increased by 30 s to 8 min as the final interval of carbofuran-AChE preincubation in the next measurements. This response was measured after each interval of preincubation time. Measurements were performed in triplicate on an Evolution 201 benchtop spectrophotometer (Thermo Fisher Scientific, Watham, MA, USA). The standard wavelength for the assay was 412 nm. The reaction was monitored continuously at 5 min intervals.

(b) Determination of carbofuran by the Ellman assay.

The 25 μmol/L carbofuran assay in 2-propanol was diluted in two series in a calibration range of concentration 0.1–25 μmol/L. Therefore, the final concentrations of carbofuran in the cuvettes were 2.4–625 nmol/L. A total of nine calibration solutions were prepared. The prepared solutions were replaced with 2-propanol and added to the reaction mixture for the Ellman AChE assay. Measurements were performed in triplicate using the Evolution 201, Spectrovis Plus, and OS3P. In the case of absorption photometers, the reaction was monitored continuously at a 5 min interval, then the difference in absorbance at a given time was monitored.

### 2.10. Acquiring of the Raw Data from OS3P

A liquid solution in a cuvette was placed in the space between the TSL230R sensor and RBG LED module (see Figure 3). As the coloration intensity of the solutions increases, the response of the sensor output is changed in the opposite direction.

**Figure 5:**
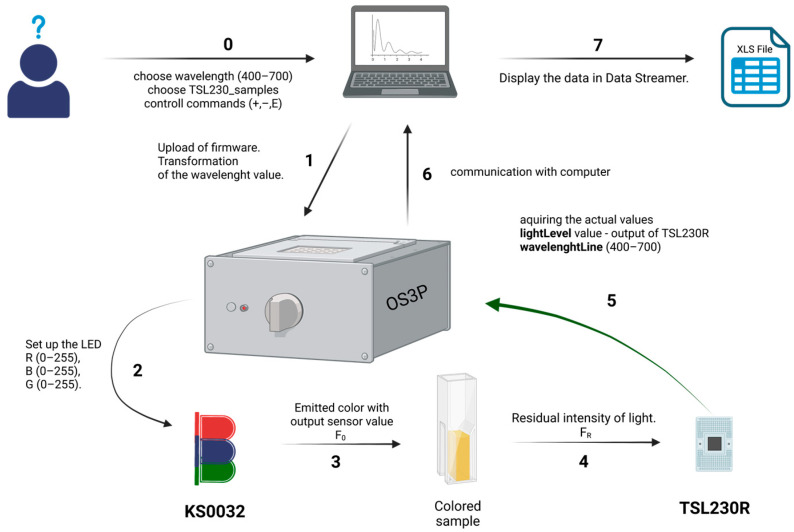
Data acquisition procedure with Open-source portable photometric platform (OS3P). The analysis algorithm is shown and each of the consecutive steps is numbered. Created with BioRender.com.

The Excel Data Streamer add-in was used to acquire the data. The desired values of ‘lightLevel’, ‘wavelengthLine’, ‘lightIntensity’ of the light source are ready to be collect immediately after installing and connecting the OS3P to the computer and confirming communication with the program. The data was collected and displayed in separate columns according to the program’s specifications. In the OS3P.ino program, the values are specified to be comma separated, so the dataset is fully compatible with the MS Excel interface and there is no need to modify the Data Streamer in any way through the Visual Basic environment in which the add-in is designed. The diagram of the data acquisition procedure with the OS3P is shown in Figure 5.

### 2.11. Statistical Evaluation of Acquired Data

(a) Standard spectrophotometry

The absorbance values at the beginning of the reaction were subtracted from the absorbance value after the observation time, and this value was used to construct a saturation curve (different concentrations of substrate vs. optical density after observation time; Michaelis–Menten non-linear model was used to evaluate the enzymatic reaction); the calibration curve of carbofuran inhibitor was obtained by comparing the values of reached optical density obtained with carbofuran added to the reaction mixture at different concentrations.

(b) The OS3P photometry

The mean value of the output frequency was calculated from at least ten to one hundred values of ‘lightLevel’ obtained from each calibrator. The final mean and standard deviation were calculated from these means. The final mean values were used for the linear evaluation of color solutions or non-linear evaluation of AChE activity in the endpoint regime for determination of enzyme activity. The ‘lightLevel’ values are described as an output frequency expressed in Hz, which is the signal collected by the TSL230R sensor and processed by the OS3P.ino firmware.

## 3. Results and Discussion

This paper describes an Open-source portable photodetection platform (OS3P), a prototype of a photometer based on Arduino UNO R3 integrated with a color-programmable RGB LED as a light source, and a TSL23OR as a light-frequency sensor [47]. The described photometer can be connected to any computer, and data can be collected using MS Excel software. The user can fully control the photometer through the Data Streamer communication line using pre-programmed commands in the OS3P.ino program [47]. This means that the wavelength for the desired color, the number of samples, or even turning the light on/off can be performed in the Data Streamer while streaming.

- Hardware and software

**Figure 6:**
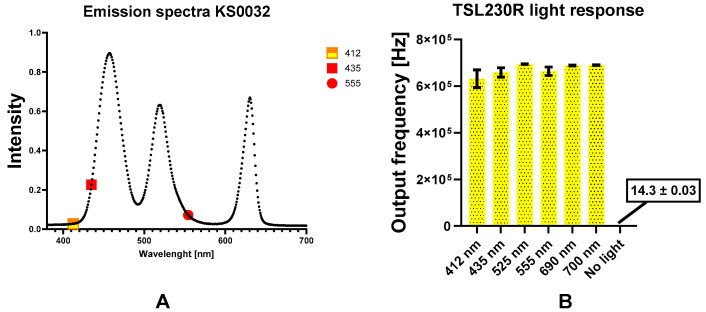
(**A**) Emission spectra of the KS0032 RGB LED module. (**B**) Comparison of the response of the TSL230R to different light conditions. The error bars in B indicate the standard deviation for n = 10 (no light) and n = 30 (with the light source was turned on).

The maximum and minimum response was obtained for colored solutions and the enzymatic assay by emitting a complementary color (See Figure 6A,B). The response of the detector under dark conditions was also collected. The average response of 16 Hz was obtained after ten repetitions. However, the number of samples for a single response had to be reduced in order to measure at least 10 values per given time. However, the time required to measure the response in dark conditions exceeded 20 min. Thus, we can conclude that parasitic light occurrence was prevented, and we can neglect this effect. We are led to this conclusion by the fact that the range of values in the calibration series of solutions with an absorbance from 0 to 2.5 is in the order of hundreds of thousands of units.

The response rate and output response of the system depend on the amount of light hitting the sensor. The operator can control the response speed of the OS3P output by setting the ‘TS230_ samples’ value. It is recommended to have high sensitivity (see the program [47]) and then to use sampling to control the speed of data acquisition. It depends mainly on the light conditions around the photometer. Sampling values higher than 20,000 are not recommended. It should be noted that, due to the Lambert–Beer law and the principle of the light sensor, there is a relatively large response delay when measuring very high concentrated solutions. For example, when measuring colored solutions, it is a good idea to select the concentration range and measure the peak concentration first. This is to prevent the sensor from getting its first response after 20–40 s. This has happened, for example, when measuring very colored solutions while the ‘TS230_ samples’ value is higher than 20,000. This is the reason to set the samples at a value of around 1000–20,000. The response rate will be steady and relatively fast (at least 5 values/1 s).

It can be seen in Figure 6A that the peak light intensities are different, which may be due to different characteristics of each diode in the module and the combination of the programmed parameters. The emission spectrum shows 3 peaks with maxima at 458, 520, and 630 nm. The full width at half maximum (FWHM, [19]) is 33, 27, and 17 nm, respectively.

According to the specifications, the sensor response should be highest at the boundary of the visible and infrared spectra [44]. RGB module will produce a response of at least 50% by emitting a color similar to the wavelengths of 450 and 950 nm.

Although there is a suggestion that the RGB LED cannot be used efficiently at wavelengths less than 510 nm [48], the OS3P has been shown to be a viable option for analysis using the Ellman method via the color-imitated wavelength of maximum absorption at 412 nm.

- Determination of Colored Solutions

**Figure 7:**
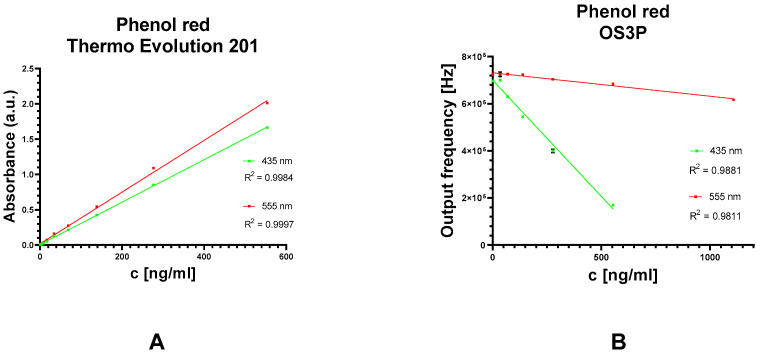
Phenol red calibration curve in the wavelength mode. Comparison between benchtop analyzer (**A**) and OS3P (**B**). Error bars indicate standard deviation for n = 3 (**A**); n = 10 (**B**).

The calibration curve for phenol red was constructed as shown in Figure 7. Two detection modes were selected, a wavelength of the color corresponding to 435 nm and 555 nm. This is the maximum absorbance obtained by the Evolution 201 benchtop analyzer. However, it was found that phenol red content in a solution with a pH of about 7.4 was best monitored at 435 nm with the OS3P system. The Evolution 201 had better performance at 555 nm. The reason for this could be that the RGB module emits light more intensely (as can be seen in Figure 6A) while mimicking the color at 435 nm instead of 555 nm. Since the TSL230R has better performance at 555 nm, another reason could be on the detector side. The limits of detection (LOD) are shown in Table 1 below. The LOD obtained with the Thermo Evolution 201 at 435 nm was 2.5 times lower than at 555 nm. The LOD for Spectrovis Plus is almost twice the difference, and the difference in LOD using OS3P is a few percent. The fact that the color-imitated 435 nm has a better performance with the tested phenol red solution is due to the higher transmittance of the color-imitated 555 nm solution. This assertion was supported by the results of the measurement of phenol red at different pH values. At pH = 7.4, the initial value of the output frequency of the OS3P with 555 nm color was higher. Therefore, the transmittance was lower and the linear calibration has a lower absolute value of the slope. However, this shows that the RGB module has limits in the emission of accurate wavelength because the result of Thermo Evolution, such as the etalon, was a better color similar to the 555 nm wavelength. It is noteworthy that the performance of the OS3P is linear in both regimes.

- AChE substrate concentration optimization

A saturation curve of the dependence of the substrate concentration and the response of the analytical system in terms of output frequency was constructed as a standard response of OS3P. In the case of the Evolution 201 benchtop spectrophotometer, the difference in absorbance for a given reaction time was observed.

**Figure 8:**
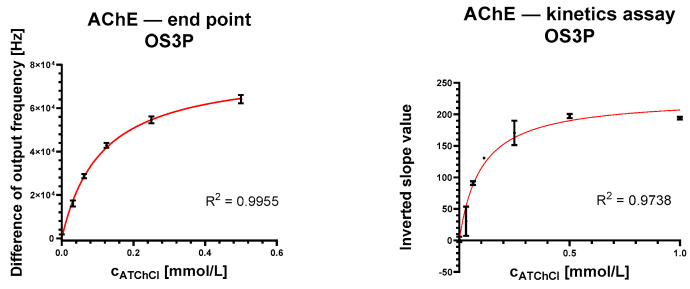
Saturation curves of acetylcholinesterase. Measurement using OS3P. The error bars indicate the standard error for n = 3.

**Figure 9:**
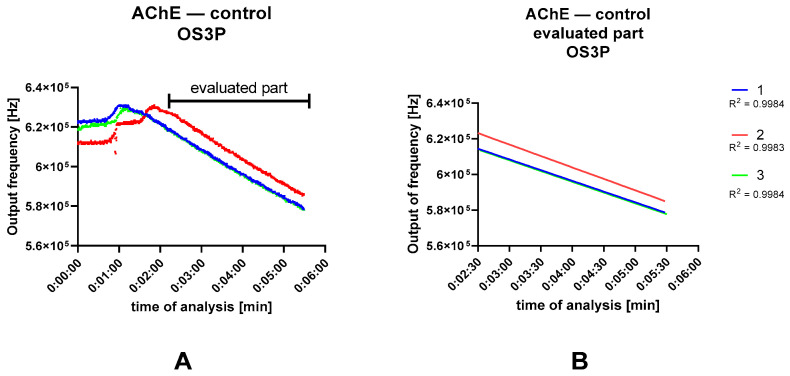
Real-time monitoring of cholinesterase activity. (**A**) Standard response of the enzyme assay. Evaluated part of the curves used to determine the kinetic parameters (**B**).

In the case of OS3P, two approaches were tested. First, subtracting the output frequency value after a given time from the value at the start of the reaction that is closest to the standard reading was considered. Because of the different onset of the linear trend found during live monitoring of the OS3P response, the difference in the linear trend over time was evaluated. The saturation curves resulting from these different assessments are shown in Figure 8. The statistical evaluation of the saturation curve is shown in Table 2. After isolating the linear segment from the initial rise, the linear trend lines were determined. Figure 9 shows an example of signal divergence of three AChE controls with the addition of pure 2-propanol.

In Figure 9A, it can be seen that during the measurement there was an initial increase in the output frequency, but after some time there was a steady linear decrease in the output frequency, which corresponds to the activity of cholinesterase. For the evaluation of the reaction kinetics, the marked section was used, or the data from 2:30 after the start of the observation were always used. When this condition was met, the resulting trends were compared using GraphPad without regard to the constant term of the linear relationship (Figure 9B). The section after the start of the data stream is characterized by an increase in sensor response, which was also observed in solutions containing inhibitors where there was no such difference between the initial and final value of the response. This artifact may be caused by droplets adhering to the cuvette wall when the reaction is initiated by substrate application. A simple solution to this situation is to delete the rising part of the results so that the linear trend is not disturbed by this artifact.

- Optimization of the Binding Time of the Inhibitor to AChE

**Figure 10:**
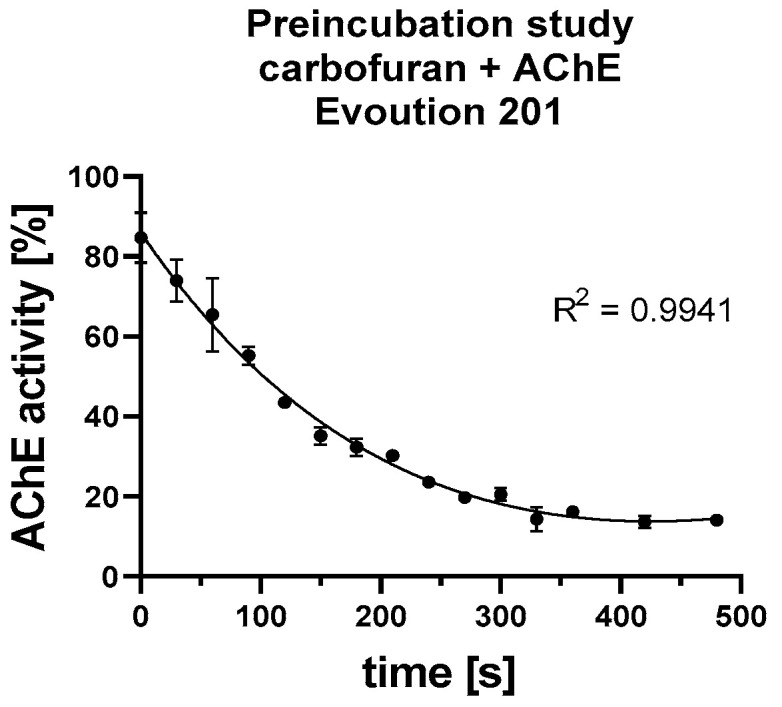
The optimal incubation time of carbofuran with AChE was measured using the Evolution 201 benchtop spectrophotometer to optimize the preincubation time of the inhibitor.

Before determining the cholinesterase inhibitor, the optimal time for the inhibitor to fully bind must be determined. The optimal time for pre-incubation of the carbamate inhibitor carbofuran was investigated. The measurement of AChE activity on Evolution 201 with different incubation times with inhibitor before starting the reaction is shown in Figure 8. Carbofuran was found to bind to AChE to achieve 10–15% residual AChE activity in 7 min (see Figure 10). Therefore, this time was used for further analysis using Evolution 201 and to refine the time required for potential determination of carbamate-type inhibitors under field conditions using the OS3P. The total analysis time was 12 min (7 min for inhibitor preincubation time, 5 min for enzyme assay observation). Sample application is not included in the time interval.

- Carbofuran assay

**Figure 11:**
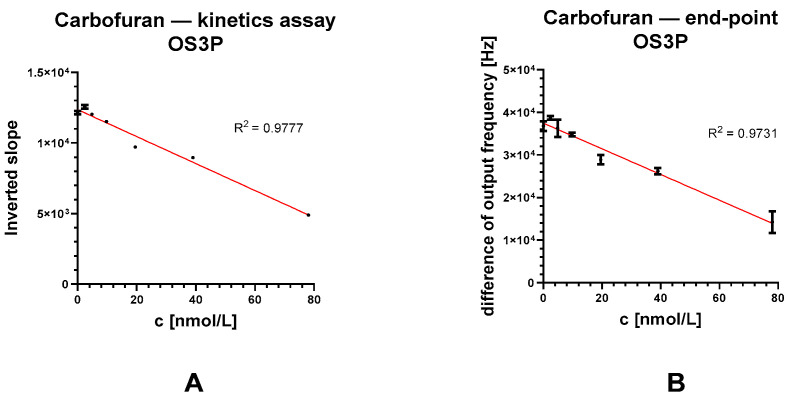
Calibration curves of carbofuran. The inverted slope values (**A**) and the difference in output frequency (**B**) were obtained and evaluated as separate markers of residual AChE activity.

Carbofuran was used as a model compound for the cholinesterase inhibitor. The calibration curve for carbofuran was constructed (see Figure 11). LODs for the detection of carbofuran were calculated from linear regression curves of the difference in slope values and the difference in subtracted output frequency values (see Table 3). In the case of endpoint analysis, LOD values of 33.5 and 13.5 nmol/L were obtained for Spectrovis Plus and the OS3P, while LOD values of 3.90 and 6.32 nmol/L were obtained for kinetic monitoring.

Kostelnik et al. published the LOD of carbofuran, 20 nmol/L, and the preincubation time, 10 min [49]. Wei et al. presented a biosensor with LOD 5 nmol/L of carbofuran using a fluorescence assay with AChE, choline-oxidase and quantum dots [50]. Another electrochemical biosensor prepared by Jeyapragasam et al. has an LOD of 3.6 nmol/L and a minimum preincubation time with carbofuran of 15 min [51]. Qu et al. presented an electrochemical sensor with a LOD for carbofuran of 0.4 nmol/L, and the required time for preincubation was 9 min [52]. Compared to these results, the OS3P requires a similar time and has a very similar performance.

The determination of cholinesterase inhibitors is the main intended application of this open-source system. This work proves that the OS3P could be used for Ellman assay, and thanks to its dimension, it can be taken outside for field testing. Although carbofuran is a banned substance, there is proof that it was still used. There is evidence of the use of carbofuran in the Canary Islands, where it has been found in nature and measured in bait obtained from the Internet. It should be noted that the Canary Islands is a place with many protected areas, so one would assume that these pesticides would be controlled there, but apparently they were not [12]. In eastern Poland, carbofuran was found in the liver of more than half of the common buzzards and a third of the eagles tested during a long-term study between 2008 and 2019. The cause of this poisoning is likely to be the illegal control of wild foxes by using baits with carbofuran or similar pesticides [11,39].

Therefore, a valid reason to introduce additional methods for testing pesticides in the environment is undoubtedly relevant. Paper dipsticks for single-use measurement of cholinesterase could be an emerging tool for screening of toxic substances [53]. These are based on the Ellman method and use the human eye for detection in comparison with colored etalon on the dipstick. It serves as a qualitative detector of cholinesterase activity. Colorimetric dipsticks based on pH changes have also been published [38,39]. However, as these dipsticks could be quantified using a smartphone or other camera, the intensity of RGB channels in the analyzed photo is still between 0 and 255. The same limit applies to the use of another massive analysis platform for photogrammetry enzyme assay [54]. In one work, these approaches have been combined to an innovative dipstick reader that can be mounted on the smartphone and reads the light intensity on the front ambient light sensor of the smartphone [40]. Despite all its advantages, the dipstick is a disposable tool. Photometers could be sustainable and affordable analyzers due to known construction and easy reproduction. However, there are few of them highlighted in this work; some of them are more expensive than the Spectrovis Plus. For example, the cost of the ready-to-use system Spectrovis Plus is the same as the sensors in the valuable work of Laganovska [55]. The OS3P could cost 1/10 of this, or even less if the user chose to buy a generic board with the same architecture.

On the other hand, another outstanding work by Hoang et al. shows the low-cost option for pollutant testing. Although the mentioned system contains similar electronic elements, the control program is different and despite the promising results; it must be noted that the biochemical reaction has not been tested and it is not an open-source device [17]. It is noteworthy that if there is an open-source system, it has been tested with end-point photometry and for use as a stand-alone photometer [16].

The use of open-source code has several important advantages. A comprehensive review of single-board architectures and their applications has recently been published [56]. On the one hand, open-source philosophy allows rapid expansion among the community, which speeds up the feedback, and on the other hand, the community can freely improve, modify, and use this platform for their specific needs. It has been proven that additional modifications and further development of the tools and technical modifications of this project by the public are possible. With this system, clear solutions of different colors can be measured as well as with commercially available analyzers, such as the Ellman assay, to determine cholinesterase activity.

Screening of alkaloids in herbs could be performed as a second possible application besides field testing of pesticides using the proposed cholinesterase assay. Indole alkaloids, e.g., in *Mitragynina speciosa*, also known as kratom, which contain mitragynine and 7-hydroxymitragynine, potential ChE inhibitors [57], as major constituents [58]. Detection of indole alkaloids, which may allow on-site monitoring of extract content [59]. Since ChEs also react with commonly used pesticides, it is necessary to verify their use before determining the alkaloid content. At the same time, it should be noted that the permitted concentration of pesticides is much lower than that of the main representatives of indole alkaloids in plants. However, the sample preparation of herbal extract could separate these interferences during pesticide screening.

## 4. Conclusions

A new photometric system, the Open-source portable photodetection platform, has been prepared based on an Arduino UNO R3, the RGB LED module KS0032, and the TSL230R light-frequency sensor. This system provides the ability to change the RGB LED light to the desired color depending on the combination of RGB coordinates to produce a color that imitates the desired wavelength. Different programmed colors were tested, and phenol red was used as a model compound to measure the analytical parameters of the proposed system. This photometer was mainly prepared for the cholinesterase assay under field conditions and point-of-care testing to detect pathological conditions related to changes in cholinesterase activity in biological samples. Acetylcholinesterase from *Electrophorus electricus* was chosen as the biorecognition element because of its similarity to human acetylcholinesterase [5]. An Ellman assay was performed to obtain the activity of AChE, and carbofuran was chosen as a model pesticide that could be screened by this system in the environment. The LOD obtained was similar to the limit obtained by the portable photometric system, which could be an alternative to the OS3P. These limits are comparable with previously published results. This work is the first step in the construction of a specialized system containing immobilized enzymes as the biorecognition element and open-source electronics as the transducer element. Although carbofuran was banned more than a decade ago, it still is being misused. Therefore, new approaches to detect this pesticide are still needed, and efforts should not be stopped.

## Figures and Tables

**Table 1:** Overview of phenol red detection limits. OS3P**—Open-source portable photodetection platform**.

Phenol Red—Limit of Detection [ng/mL]			
Analyzer	Thermo Evolution 201	Spectrovis Plus	OS3P
Color appropriate for 435 nm	10.7	53.4	73.1
Color appropriate for 555 nm	25.2	91.2	81.3

**Table 2:** Comparison of the results from the new platform and the benchtop photometer.

Analyzer	Thermo Evolution 201		Open-Source Portable Photodetection Platform	
	K_M_ [mmol/L]	r^2^	K_M_ [mmol/L]	r^2^
End-point assay	0.116	0.994	0.110	0.996
Kinetic assay			0.0982	0.974

**Table 3:** Comparison of the detection limits of carbofuran based on the methods and analyzers used.

Limits of Detection—Carbofuran (nmol/L)		
Analyzer	Spectrovis Plus	OS3P
Inverted slope values	3.88	6.32
End-point Assay	33.5	13.5

## Data Availability

Not applicable.

## References

[1] Pundir C.S., Malik A., Preety (2019). Bio-sensing of organophosphorus pesticides: A review. Biosens. Bioelectron..

[2] Jain U., Saxena K., Hooda V., Balayan S., Singh A.P., Tikadar M., Chauhan N. (2022). Emerging vistas on pesticides detection based on electrochemical biosensors—An update. Food Chem..

[3] Liu D.M., Xu B.J., Dong C. (2021). Recent advances in colorimetric strategies for acetylcholinesterase assay and their applications. Trac. Trend. Anal. Chem..

[4] Pope C.N., Brimijoin S. (2018). Cholinesterases and the fine line between poison and remedy. Biochem. Pharmacol..

[5] Choi R.J., Roy A., Jung H.J., Ali M.Y., Min B.-S., Park C.H., Yokozawa T., Fan T.-P., Choi J.S., Jung H.A. (2016). BACE1 molecular docking and anti-Alzheimer’s disease activities of ginsenosides. J. Ethnopharmacol..

[6] Goud K.Y., Teymourian H., Sandhu S.S., Tostado N., Mishra R.K., Moore L.C., Harvey S.P., Wang J. (2020). OPAA/fluoride biosensor chip towards field detection of G-type nerve agents. Sens. Actuators B Chem..

[7] Pundir C.S., Chauhan N. (2012). Acetylcholinesterase inhibition-based biosensors for pesticide determination: A review. Anal. Biochem..

[8] Cao J., Wang M., Yu H., She Y., Cao Z., Ye J., Abd El-Aty A.M., Hacimuftuoglu A., Wang J., Lao S. (2020). An Overview on the Mechanisms and Applications of Enzyme Inhibition-Based Methods for Determination of Organophosphate and Carbamate Pesticides. J. Agric. Food Chem..

[9] Pohanka M. (2011). Cholinesterases, a target of pharmacology and toxicology. Biomed. Pap. Med. Fac. Univ. Palacky Olomouc Czechoslov..

[10] Sarkar B., Alam S., Rajib T.K., Islam S.S., Araf Y., Ullah M.A. (2021). Identification of the most potent acetylcholinesterase inhibitors from plants for possible treatment of Alzheimer’s disease: A computational approach. Egypt. J. Med. Hum. Genet..

[11] Kitowski I., Łopucki R., Stachniuk A., Fornal E. (2020). A pesticide banned in the European Union over a decade ago is still present in raptors in Poland. Environ. Conserv..

[12] Ruiz-Suarez N., Boada L.D., Henriquez-Hernandez L.A., Gonzalez-Moreo F., Suarez-Perez A., Camacho M., Zumbado M., Almeida-Gonzalez M., Del Mar Travieso-Aja M., Luzardo O.P. (2015). Continued implication of the banned pesticides carbofuran and aldicarb in the poisoning of domestic and wild animals of the Canary Islands (Spain). Sci. Total Environ..

[13] de Siqueira A., Salvagni F.A., Yoshida A.S., Goncalves-Junior V., Calefi A.S., Fukushima A.R., Spinosa Hde S., Maiorka P.C. (2015). Poisoning of cats and dogs by the carbamate pesticides aldicarb and carbofuran. Res. Vet. Sci..

[14] Zeljezic D., Vrdoljak A.L., Radic B., Fuchs N., Berend S., Orescanin V., Kopjar N. (2007). Comparative evaluation of acetylcholinesterase status and genome damage in blood cells of industrial workers exposed to carbofuran. Food Chem. Toxicol..

[15] Di Nonno S., Ulber R. (2022). Portuino—A Novel Portable Low-Cost Arduino-Based Photo- and Fluorimeter. Sensors.

[16] Anzalone G.C., Glover A.G., Pearce J.M. (2013). Open-source colorimeter. Sensors.

[17] Hoang L.Q., Chi H.B.L., Khanh D.N.N., Vy N.T.T., Hanh P.X., Vu T.N., Lam H.T., Phuong N.T.K. (2021). Development of a low-cost colorimeter and its application for determination of environmental pollutants. Spectrochim. Acta Part A Mol. Biomol. Spectrosc..

[18] Machado M.C., Vimbela G.V., Tripathi A. (2022). Creation of a low cost, low light bioluminescence sensor for real time biological nitrate sensing in marine environments. Environ. Technol..

[19] Kurata K. (2021). Open-source colorimeter assembled from laser-cut plates and plug-in circuits. Hardwarex.

[20] Fu Q., Wu Z., Xu F., Li X., Yao C., Xu M., Sheng L., Yu S., Tang Y. (2016). A portable smart phone-based plasmonic nanosensor readout platform that measures transmitted light intensities of nanosubstrates using an ambient light sensor. Lab Chip.

[21] Arafat Hossain M., Canning J., Ast S., Cook K., Rutledge P.J., Jamalipour A. (2015). Combined “dual” absorption and fluorescence smartphone spectrometers. Opt. Lett..

[22] Bergua J.F., Alvarez-Diduk R., Idili A., Parolo C., Maymo M., Hu L., Merkoci A. (2022). Low-Cost, User-Friendly, All-Integrated Smartphone-Based Microplate Reader for Optical-Based Biological and Chemical Analyses. Anal. Chem..

[23] Jeong H., Shin S., Hwang J., Kim Y.-J., Choi S. (2021). Open-Source Fluorescence Spectrometer for Noncontact Scientific Research and Education. J. Chem. Educ..

[24] Arduini F., Cinti S., Caratelli V., Amendola L., Palleschi G., Moscone D. (2019). Origami multiple paper-based electrochemical biosensors for pesticide detection. Biosens. Bioelectron..

[25] Caratelli V., Fegatelli G., Moscone D., Arduini F. (2022). A paper-based electrochemical device for the detection of pesticides in aerosol phase inspired by nature: A flower-like origami biosensor for precision agriculture. Biosens. Bioelectron..

[26] Bilal S., Sami A.J., Hayat A., Fayyaz Ur Rehman M. (2022). Assessment of pesticide induced inhibition of *Apis mellifera* (honeybee) acetylcholinesterase by means of N-doped carbon dots/BSA nanocomposite modified electrochemical biosensor. Bioelectrochemistry.

[27] Ding J., Li B., Chen L., Qin W. (2016). A Three-Dimensional Origami Paper-Based Device for Potentiometric Biosensing. Angew. Chem. Int. Ed. Engl..

[28] Mishra R.K., Hubble L.J., Martin A., Kumar R., Barfidokht A., Kim J., Musameh M.M., Kyratzis I.L., Wang J. (2017). Wearable Flexible and Stretchable Glove Biosensor for On-Site Detection of Organophosphorus Chemical Threats. ACS Sens..

[29] Thet Tun W.S., Saenchoopa A., Daduang S., Daduang J., Kulchat S., Patramanon R. (2023). Electrochemical biosensor based on cellulose nanofibers/graphene oxide and acetylcholinesterase for the detection of chlorpyrifos pesticide in water and fruit juice. Rsc. Adv..

[30] Tao H., Liu F., Ji C., Wu Y., Wang X., Shi Q. (2022). A novel electrochemical sensing platform based on the esterase extracted from kidney bean for high-sensitivity determination of organophosphorus pesticides. Rsc. Adv..

[31] Fuyal M., Giri B. (2020). A Combined System of Paper Device and Portable Spectrometer for the Detection of Pesticide Residues. Food Analytical. Methods.

[32] Bueno D., Alonso G., Muñoz R., Marty J.L. (2014). Low-cost and portable absorbance measuring system to carbamate and organophosphate pesticides. Sens. Actuators B Chem..

[33] Gong C., Fan Y., Zhao H. (2022). Recent advances and perspectives of enzyme-based optical biosensing for organophosphorus pesticides detection. Talanta.

[34] Ellman G.L., Courtney K.D., Andres V., Feather-Stone R.M. (1961). A new and rapid colorimetric determination of acetylcholinesterase activity. Biochem. Pharmacol..

[35] Eyer P., Worek F., Kiderlen D., Sinko G., Stuglin A., Simeon-Rudolf V., Reiner E. (2003). Molar absorption coefficients for the reduced Ellman reagent: Reassessment. Anal. Biochem..

[36] Pohanka M., Hrabinova M., Kuca K., Simonato J.P. (2011). Assessment of acetylcholinesterase activity using indoxylacetate and comparison with the standard Ellman’s method. Int. J. Mol. Sci..

[37] Keresteš O., Pohanka M. (2022). Enzymatic Biosensors for the Environmental Analysis of Pesticides. Chem. Listy.

[38] Pohanka M., Karasova J.Z., Kuca K., Pikula J., Holas O., Korabecny J., Cabal J. (2010). Colorimetric dipstick for assay of organophosphate pesticides and nerve agents represented by paraoxon, sarin and VX. Talanta.

[39] No H.Y., Kim Y.A., Lee Y.T., Lee H.S. (2007). Cholinesterase-based dipstick assay for the detection of organophosphate and carbamate pesticides. Anal. Chim. Acta.

[40] Fu Q., Zhang C., Xie J., Li Z., Qu L., Cai X., Ouyang H., Song Y., Du D., Lin Y. (2019). Ambient light sensor based colorimetric dipstick reader for rapid monitoring organophosphate pesticides on a smart phone. Anal. Chim. Acta.

[41] Pitschmann V., Matějovský L., Lunerová K., Dymák M., Urban M., Králík L. (2019). Detection Papers with Chromogenic Chemosensors for Direct Visual Detection and Distinction of Liquid Chemical Warfare Agents. Chemosensors.

[42] Pidany F., Kroustkova J., Al Mamun A., Suchankova D., Brazzolotto X., Nachon F., Chantegreil F., Dolezal R., Pulkrabkova L., Muckova L. (2023). Highly selective butyrylcholinesterase inhibitors related to Amaryllidaceae alkaloids—Design, synthesis, and biological evaluation. Eur. J. Med. Chem..

[43] BCSengage Arduino Starter Kit Base Replacement. https://www.thingiverse.com/thing:1286765.

[44] TAOS TSL230R Datasheet. https://pdf1.alldatasheet.com/datasheet-pdf/view/153439/ETC1/TSL230R.html.

[45] Ks0032 Keyestudio RGB LED Module. https://wiki.keyestudio.com/Ks0032_keyestudio_RGB_LED_Module.

[46] Meyer A. High Sensitivity Light Sensor TSL230R + Arduino. http://adam-meyer.com/arduino/TSL230R.

[47] Kerestes O., Pohanka M. Open Source Portable Photodetection Platform (OS3P).

[48] Carreres-Prieto D., García J.T., Cerdán-Cartagena F., Suardiaz-Muro J. (2020). Performing Calibration of Transmittance by Single RGB-LED within the Visible Spectrum. Sensors.

[49] Kostelnik A., Kopel P., Cegan A., Pohanka M. (2017). Construction of an Acetylcholinesterase Sensor Based on Synthesized Paramagnetic Nanoparticles, a Simple Tool for Neurotoxic Compounds Assay. Sensors.

[50] Wei J.C., Wei B., Yang W., He C.W., Su H.X., Wan J.B., Li P., Wang Y.T. (2018). Trace determination of carbamate pesticides in medicinal plants by a fluorescent technique. Food Chem. Toxicol..

[51] Jeyapragasam T., Saraswathi R. (2014). Electrochemical biosensing of carbofuran based on acetylcholinesterase immobilized onto iron oxide-chitosan nanocomposite. Sens. Actuators B Chem..

[52] Qu Y., Sun Q., Xiao F., Shi G., Jin L. (2010). Layer-by-Layer self-assembled acetylcholinesterase/PAMAM-Au on CNTs modified electrode for sensing pesticides. Bioelectrochemistry.

[53] Matejovsky L., Pitschmann V. (2018). New Carrier Made from Glass Nanofibres for the Colorimetric Biosensor of Cholinesterase Inhibitors. Biosensors.

[54] Pohanka M., Zakova J. (2021). A Smartphone Camera Colorimetric Assay of Acetylcholinesterase and Butyrylcholinesterase Activity. Sensors.

[55] Laganovska K., Zolotarjovs A., Vazquez M., Mc Donnell K., Liepins J., Ben-Yoav H., Karitans V., Smits K. (2020). Portable low-cost open-source wireless spectrophotometer for fast and reliable measurements. Hardwarex.

[56] Alvarez J.L., Mozo J.D., Duran E. (2021). Analysis of Single Board Architectures Integrating Sensors Technologies. Sensors.

[57] Innok W., Hiranrat A., Chana N., Rungrotmongkol T., Kongsune P. (2021). In silico and in vitro anti-AChE activity investigations of constituents from *Mytragyna speciosa* for Alzheimer’s disease treatment. J. Comput. Aided Mol. Des..

[58] Kikura-Hanajiri R., Kawamura M., Maruyama T., Kitajima M., Takayama H., Goda Y. (2009). Simultaneous analysis of mitragynine, 7-hydroxymitragynine, and other alkaloids in the psychotropic plant “kratom” (*Mitragyna speciosa*) by LC-ESI-MS. Forensic Toxicol..

[59] Omar F., Tareq A.M., Alqahtani A.M., Dhama K., Sayeed M.A., Emran T.B., Simal-Gandara J. (2021). Plant-Based Indole Alkaloids: A Comprehensive Overview from a Pharmacological Perspective. Molecules.

